# Renal safety of zoledronic acid with thalidomide in patients with myeloma: a pharmacokinetic and safety sub-study

**DOI:** 10.1186/1472-6904-8-2

**Published:** 2008-03-31

**Authors:** Andrew Spencer, Andrew Roberts, Nola Kennedy, Christina Ravera, Serge Cremers, Sanela Bilic, Terry Neeman, Michael Copeman, Horst Schran, Kevin Lynch

**Affiliations:** 1 Department of Haematology and Bone Marrow Transplantation, The Alfred Hospital, Prahan, Victoria, Australia; 2 Department of Haematology, Royal Melbourne Hospital, Parkville, Victoria, Australia; 3 Department of Clinical Pharmacology, Novartis Pharmaceuticals Corporation, East Hanover, New Jersey 07936, USA; 4 Covance Pty. Ltd., Canberra, ACT, Australia; 5 Novartis Pharmaceuticals Australia Pty. Ltd., 54 Waterloo Rd, North Ryde NSW 2113, Australia

## Abstract

**Background:**

Cases of impaired renal function have been reported in patients who had been treated with both zoledronic acid and thalidomide for myeloma. Hence, we conducted a safety study of zoledronic acid and thalidomide in myeloma patients participating in a trial of maintenance therapy.

**Methods:**

Twenty-four patients who were enrolled in a large randomized trial of thalidomide vs no thalidomide maintenance therapy for myeloma, in which all patients also received zoledronic acid, were recruited to a pharmacokinetic and renal safety sub-study, and followed for up to 16 months.

**Results:**

No significant differences by Wilcoxon rank-sum statistic were found in zoledronic acid pharmacokinetics or renal safety for up to 16 months in patients randomized to thalidomide or not.

**Conclusion:**

In myeloma patients receiving maintenance therapy, the combination of zoledronic acid and thalidomide appears to confer no additional renal safety risks over zoledronic acid alone.

## Background

Modern therapy of multiple myeloma (MM) combines treatments to induce disease response (e.g. thalidomide, corticosteroids and cytotoxic agents) with supportive care to prevent bone and infective complications (e.g. bisphosphonates and antibiotics). However, combining agents might cause interactions that increase toxicity or lessen efficacy. Case reports suggested that MM patients receiving zoledronic acid combined with thalidomide had higher risks of renal impairment [1]. Hence, the United States' Food and Drug Administration requested that Novartis (manufacturer of zoledronic acid) conduct a study to look for pharmacokinetic interaction between zoledronic acid and thalidomide, and to monitor renal safety of MM patients receiving both drugs.

The Australasian Leukaemia and Lymphoma Group (ALLG) was conducting a large (>200 patients), randomized clinical trial (ALLG MM6) in MM patients, who had received high-dose therapy and autologous stem cell transplantation (ASCT), to ascertain the efficacy and safety of thalidomide and prednisolone maintenance therapy compared to prednisolone maintenance therapy post-transplantation. All patients were scheduled to receive adjunctive zoledronic acid intravenously on a 4^th^-weekly basis. The ALLG agreed to incorporate a safety sub-study into the ALLG MM6 study to assess the pharmacokinetics of zoledronic acid and to monitor renal function in patients randomized to receive prednisolone and zoledronic acid alone or in combination with thalidomide, for up to 16 months.

## Methods

From two Australian hospitals (The Alfred Hospital, Melbourne and The Royal Melbourne Hospital) participating in the ALLGMM6 study, a sample of 24 patients was enrolled in this sub-study, with institutional ethical approval and informed consent. All patients entered the study 6–7 weeks after high-dose therapy and ASCT for MM. Twelve patients were randomized to Arm 1: Thalidomide (100 mg nocte for 14 days, then 200 mg nocte to end of study, if tolerated) and prednisolone (50 mg orally on alternate days) in conjunction with zoledronic acid (4 mg intravenously every 4 weeks) and 12 were randomized to Arm 2: prednisolone and zoledronic acid.

Patients' ages ranged from 41–68, with a median of 60 in Arm 1 and 57 in Arm 2. There were 7 males and 5 females in each Arm. No patient had progressive myeloma, ECOG score > 2, renal, hepatic or severe marrow impairment at study entry. All patients agreed to precautions related to teratogenicity.

Thalidomide was provided as 50 mg tablets by Pharmion Pty. Ltd. Zoledronic acid was provided as 4 mg concentrated liquid in vials, to be made up to 100 ml for intravenous infusion over 15 minutes.

For renal safety monitoring, prior to each zoledronic acid infusion, patients' serum creatinines were checked, and any adverse events potentially related to zoledronic acid, thalidomide or prednisolone were collected for up to 16 months on study.

For the pharmacokinetic study, venous blood samples (3 mL in heparinized tubes) were drawn from patients' arms contra-lateral to the site of zoledronic acid infusions or from central venous lines at the following times: Prior to the 1^st ^and 2^nd ^doses of zoledronic acid (on Days 1 and 29) and 1, 2, 4, 8, 24 and 48 hours after the 1^st ^and 2^nd ^doses. Samples were centrifuged for 15 minutes at 2000 × G, and the plasma transferred into a labelled tube that was frozen below -20C. All pharmacokinetic samples were shipped in dry ice to a central laboratory (Novartis Pharma AG, Rueil-Malmaison, France) for analysis performed by a sensitive radioimmunoassay for zoledronic acid [2].

One patient (from Arm 1) was excluded from analysis because their pre-dose sample contained zoledronic acid, and the reasons for this finding were unclear. There were also five missing samples (3 from Arm 1 and 2 from Arm 2) for post-zoledronic acid pharmacokinetic analysis on Day 29.

Non-parametric Wilcoxon rank-sum statistics were used (by Covance Pty. Ltd, Canberra, Australia) to compare non-compartmental pharmacokinetics (Cmax and AUC) of each zoledronic acid infusion in patients who did or did not also receive thalidomide. A parametric repeated-measures t-test analysis was also conducted to assess PK differences over 1^st ^and 2^nd ^infusions – although no change in plasma PK of zoledronic acid was seen with subsequent doses in an earlier (non-combination) PK study [3]. The study was powered with 12 patients in each group to have 80% chance of detecting (with p = 0.05) a 43% difference in PK of zoledronic acid in conjunction with thalidomide, based on 27% interpatient variability of Cmax for zoledronic acid in an earlier study [4].

## Results

### Pharmacokinetic analyses

**Figure 1:**
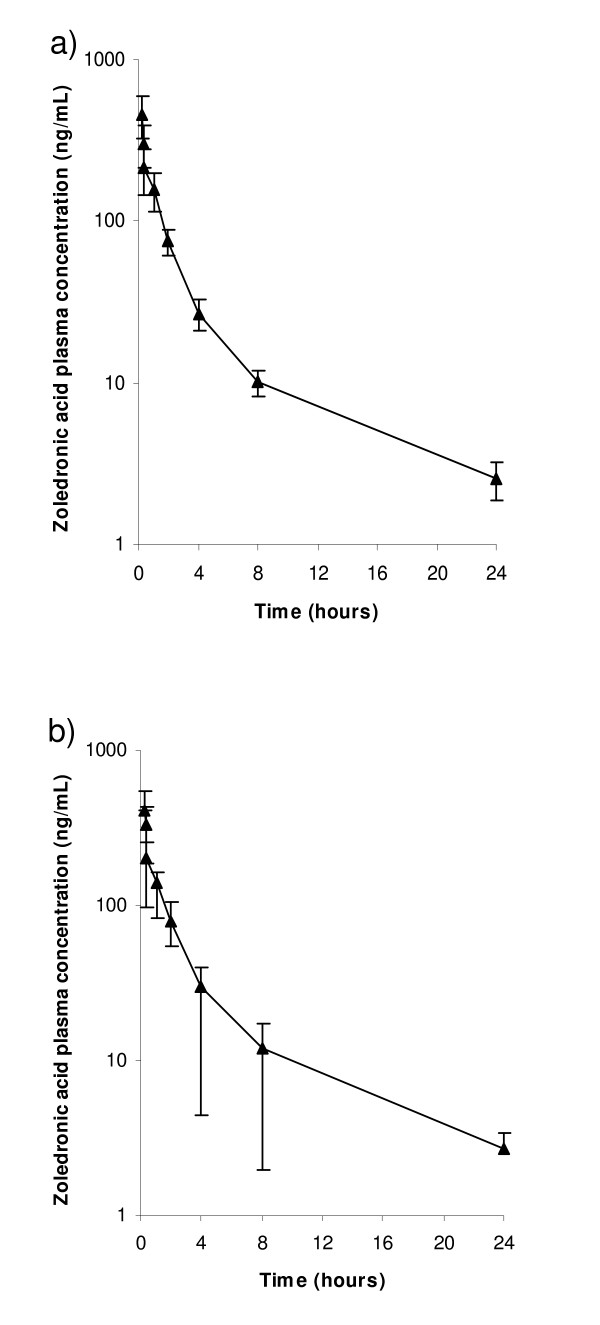
**a: Means (± standard deviation) of plasma concentrations of zoledronic acid after 1^st ^infusion in Arm1 (n = 11) patients who received zoledronic acid, thalidomide and prednisolone.** b: Means (± standard deviation) of plasma concentrations of zoledronic acid after 1^st ^infusion in Arm2 (n = 12) patients who received zoledronic acid alone.

Mean zoledronic acid plasma concentrations over 24 hours after infusion #1 in patients receiving zoledronic acid in combination with thalidomide and prednisolone (Arm 1; n = 11) or with prednisolone alone (Arm 2; n = 12) are shown in Figure 1. Mean Cmax after infusion #1 was 462 (± 135) ng/mL in Arm 1 and 409 (± 142) ng/mL. Mean AUC(0–24 hr) was 639 (± 86) ng.h/mL in Arm 1 and 651 (± 140) ng.h/mL in Arm 2. Similar figures were obtained after infusion #2 (not shown). No differences were statistically significant by t-test (p = 0.05).

### Renal safety

**Figure 2:**
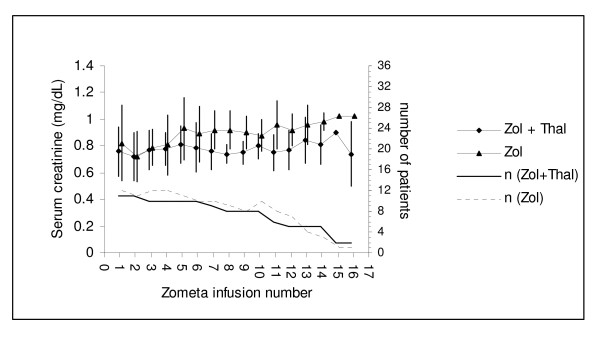
**Means (± standard deviations) of serum creatinines prior to (up to 16) infusions of zoledronic acid in patients on Arm1 (diamonds) [receiving zoledronic acid, thalidomide and prednisolone] and Arm 2 (triangles) [receiving zoledronic acid alone].** Numbers of patients receiving each infusion are also shown, by reference to the scale on the right side.

Mean serum creatinine values for up to 16 infusions of zoledronic acid are shown in Figure 2. Again no differences between patients in Arm 1 (zoledronic acid, thalidomide and prednisolone) and Arm 2 (zoledronic acid with prednisolone alone) were statistically significant. Patients' creatinine clearance (calculated from serum creatinine using Cockcroft & Gault formula) for arms 1 and 2 showed mean (SD) values of 104 +/- 27 mL/min (n = 11) and 106 +/- 27 mL/min (n = 12), respectively, at baseline and 99 +/- 20 mL/min (n = 5) and 100 +/- 20 (n = 4), respectively, after 13 infusions. The model estimated difference between arms 1 and 2, taking into account infusion number and any interaction between treatment arms and infusion, was 0.46 mL/min (P = 0.60), supporting the conclusion of no effect on renal function by the addition of thalidomide to the zoledronic acid treatment regimen. Withdrawals from the sub-study were no different between Arms 1 and 2, and largely due to progressive disease.

## Discussion

This pharmacokinetic and renal safety sub-study of the combination of zoledronic acid with thalidomide and prednisolone in MM patients shows no likely PK interaction between zoledronic acid and thalidomide (at doses up to 200 mg/day). There was also no detectable increase in serum creatinine in patients receiving combined therapy for up to 16 months, unlike in previous case reports [1].

MM patients who either a) do not receive concomitant corticosteroid therapy, b) receive higher doses of thalidomide than 200 mg/day, or c) have underlying or intercurrent renal impairment could have interactions that were not detected in this safety sub-study. Nevertheless, the absence of PK interaction or renal impairment seen in this safety sub-study is reassuring, given recent widespread prescription of both thalidomide and zoledronic acid in MM patients.

## Conclusion

In myeloma patients receiving maintenance therapy, the combination of zoledronic acid and thalidomide appears to confer no additional renal safety risks over zoledronic acid alone.

## Competing interests

Drs Spencer and Roberts have received research funds and honoraria from Novartis; Dr Copeman is a consultant to Novartis; Drs Ravera, Cremers, Bilic, Schran and Lynch are employees of Novartis, the manufacturer of zoledronic acid.

## Authors' contributions

AS, HS, MC and KL conceived the study. AS, AR and NK carried out the clinical aspects of the study. TN contributed to the statistical design of the study and carried out analyses of results. CR, SC, SB and HS carried out the pharmacokinetic analyses of zoledronic acid. MC wrote the manuscript.

## Pre-publication history

The pre-publication history for this paper can be accessed here:


